# [1,1′-Diphenyl-3,3′-(propane-1,3-diyldinitrilo)dibut-1-enolato]copper(II)

**DOI:** 10.1107/S1600536809001081

**Published:** 2009-01-17

**Authors:** Mehdi Salehi, Soraia Meghdadi, Mehdi Amirnasr, Kurt Mereiter

**Affiliations:** a Department of Chemistry, Isfahan University of Technology, Isfahan 84156-83111, Iran; b Faculty of Chemistry, Vienna University of Technology, Getreidemarkt 9/164SC, A-1060 Vienna, Austria

## Abstract

The title compound, [Cu(C_23_H_24_N_2_O_2_)] or [Cu{(BA)_2_pn}], where (BA)_2_pn is 1,1′-diphenyl-3,3′-(propane-1,3-diyldinitrilo)dibut-1-enolate, is a mononuclear copper(II) complex, located on a twofold axis. The four-coordinate Cu^II^ atom is in a tetra­hedrally distorted square plane defined by the N and O atoms of the Schiff base ligand. In the tetra­dentate ligand, the two chelate rings are twisted relative to each other, making a dihedral angle of 36.57 (3)°.

## Related literature

For general background, see: Bunce *et al.* (1998 ▶); Klement *et al.* (1999 ▶); Meghdadi *et al.* (2008 ▶); Mikuriya *et al.* (2002 ▶); Filomeni *et al.* (2007 ▶). For a structure determination of the title compound in space group *Cc*, see: Sarkar *et al.* (2008 ▶). For the structure of a polymorph, see: Arıcı (2006 ▶). For related structures, see: Arıcı *et al.* (2001 ▶); Dehghanpour *et al.* (2005 ▶).
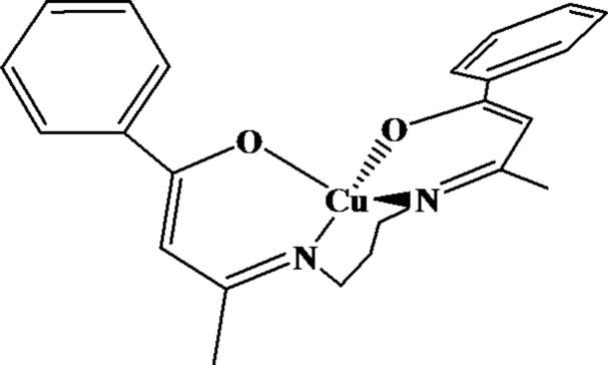


## Experimental

### 

#### Crystal data

[Cu(C_23_H_24_N_2_O_2_)]
*M* *_r_* = 423.98
Monoclinic, 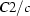

*a* = 12.2047 (12) Å
*b* = 20.320 (2) Å
*c* = 8.9992 (9) Å
β = 117.405 (1)°
*V* = 1981.3 (3) Å^3^
*Z* = 4
Mo *K*α radiation
μ = 1.12 mm^−1^
*T* = 100 (2) K
0.50 × 0.46 × 0.35 mm

#### Data collection

Bruker APEXII CCD diffractometer
Absorption correction: multi-scan (*SADABS*; Bruker, 2008 ▶) *T*_min_ = 0.57, *T*_max_ = 0.68
11028 measured reflections
2881 independent reflections
2780 reflections with *I* > 2σ(*I*)
*R*_int_ = 0.019

#### Refinement

*R*[*F* ^2^ > 2σ(*F* ^2^)] = 0.022
*wR*(*F* ^2^) = 0.063
*S* = 1.05
2881 reflections
129 parameters
H-atom parameters constrained
Δρ_max_ = 0.42 e Å^−3^
Δρ_min_ = −0.34 e Å^−3^

### 

Data collection: *APEX2* (Bruker, 2008 ▶); cell refinement: *SAINT* (Bruker, 2008 ▶); data reduction: *SAINT*; program(s) used to solve structure: *SHELXS97* (Sheldrick, 2008 ▶); program(s) used to refine structure: *SHELXL97* (Sheldrick, 2008 ▶); molecular graphics: *SHELXTL* (Sheldrick, 2008 ▶); software used to prepare material for publication: *SHELXTL*.

## Supplementary Material

Crystal structure: contains datablocks I, global. DOI: 10.1107/S1600536809001081/dn2424sup1.cif

Structure factors: contains datablocks I. DOI: 10.1107/S1600536809001081/dn2424Isup2.hkl

Additional supplementary materials:  crystallographic information; 3D view; checkCIF report

## Figures and Tables

**Fig. 1.**
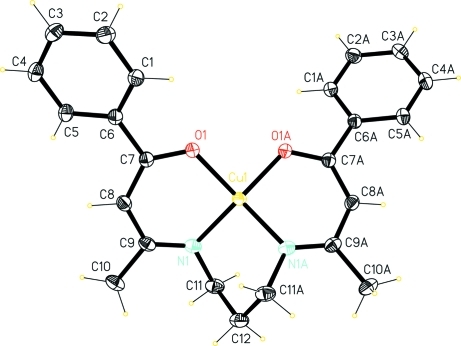
The ORTEP drawing of (I), with atom labeling scheme. Displacement ellipsoids are drawn at the 50% probability level. [Symmetry code: (A) 1-x, y, 1/2-z].

## supplementary crystallographic information

### Comment

The chemistry of copper(II) complexes with N_2_O_2_ Schiff base ligands have been investigated extensively in recent years owing to their interesting physico–chemical properties, and their potential application in catalytic reactions and biological activities (Bunce *et al.*, 1998; Klement *et al.*; 1999, Filomeni *et al.*, 2007). These complexes are also attractive because of their preparative accessibility, diversity and structural variability. Most of the earlier investigations were carried out on the tetradentate Schiff base ligand salen and its derivatives, while *N*,*N*'–bis(benzoylacetone)–1,3–propylenediimine and its derivatives have rarely been studied [Mikuriya *et al.* 2002]. Two alternative reports on the synthesis and structure of Cu(II) complexes with (BA)_2_pn ligand under different conditions is already published [Arıcı, 2006; Sarkar *et al.*, 2008]. In this context, we herein report the synthesis and structure of the title compound, [Cu((BA)_2_pn)], (I), and make a brief comparison with the reported structures.

The structure of [Cu((BA)_2_pn)], (I), is shown in Fig. 1. The complex has symmetry *C*_2_ with Cu(1) and C(12) on a *C*_2_ axis. The coordination geometry around Cu(II) center may be described as distorted square planar. This is indicated by the chelate angles which deviate from 90° (90.19 (4)° (O(1)–Cu(1)–O(1)^i^) to 94.56 (3)° (O(1)–Cu(1)–N(1))) and 180° (154.41 (4)° for O(1)–Cu(1)–N(1)^i^ = O(1)^i^–Cu(1)–N(1)). The dihedral angle between the two Cu–O,N chelate rings is 36.57 (3)°. The Cu–O and Cu–N distances of the coordinated (BA)_2_pn in the equatorial plane, Cu(1)–O(1) = Cu(1)–O(1)^i^ = 1.9145 (7) Å, Cu(1)–N(1) = Cu(1)–N(1)^i^ = 1.9394 (9) Å, compare well with the Cu–O and Cu–N distances found in the related complex [Cu((BA)_2_en)] [Cu–O_av_ = 1.913 (2) Å, Cu–N_av_ = 1.930 (3) Å; Dehghanpour *et al.*, (2005)]. The crystal structure is, in principle, identical to that reported by Sarkar *et al.*, 2008. The space group was determined by these authors as *Cc*, however, it was overlooked that the structure has *C*_2_ axes parallel to *b* and therefore adopts space group symmetry *C*2/*c*. Due to this overlooked space group symmetry (*Cc* instead of *C*2/*c*), chemically equivalent bond lengths and angles in the reported structure vary widely (bond length differences of chemically equivalent bonds up to 0.17 Å (C19–C20 and C8–C9)). In another report (Arıcı, 2006), a polymorphic form of this compound with space group *C*2/*c* and a molecular conformation which differs most significantly from Sarkar's and the present work is presented. Among other features, this complex shows a more planar coordination of Cu with *transoid* O–Cu–N angles of about 170° and a half-chair conformation for the Cu–N,N chelate ring, whereas (I) exhibits a twist-boat conformation similar to the dimethyl-substituted homologue (at C(12) of (I)) described by Arıcı *et al.* (2001).

### Experimental

H_2_(BA)_2_pn, *N*,*N*'–bis(benzoylacetone)–1,3–propylenediimine was prepared according to the literature [Meghdadi *et al.* 2008] by the condensation of of benzoylacetone (0.324 g, 2 mmol) with 1,3–propylenediamine (0.074 g, 1 mmol) in methanol at room temperature. To a solution of H_2_(BA)_2_pn (0.362 g, 1 mmol) in methanol (30 ml) was added with stirring copper(II) acetate monohydrate (0.200 g, 1 mmol). The mixture was stirred at room temperature for about 30 min and then filtered. The filtrate was kept undisturbed in air and black oval–shaped crystals of (I) were formed upon slow evaporation of the solvent after two days. The crystals were filtered off, washed with cold methanol and dried under vacuum.

### Refinement

All H atoms attached to C atoms were fixed geometrically and treated as riding with C—H = 0.95Å (aromatic), 0.99Å (methylene) and 0.98Å (methyl) with *U*_iso_(H) = 1.2 *U*_eq_(aromatic, methylene) or *U*_iso_(H) = 1.5 *U*_eq_(methyl).

### Crystal data

**Table:**

[Cu(C_23_H_24_N_2_O_2_)]	*F*(000) = 884
*M**_r_* = 423.98	*D*_x_ = 1.421 Mg m^−^^3^
Monoclinic, *C*2/*c*	Mo *K*α radiation, λ = 0.71073 Å
Hall symbol: -C 2yc	Cell parameters from 8926 reflections
*a* = 12.2047 (12) Å	θ = 2.1–30.0°
*b* = 20.320 (2) Å	µ = 1.12 mm^−^^1^
*c* = 8.9992 (9) Å	*T* = 100 K
β = 117.405 (1)°	Oval, black
*V* = 1981.3 (3) Å^3^	0.50 × 0.46 × 0.35 mm
*Z* = 4	

### Data collection

**Table:**

Bruker APEXII CCD diffractometer	2881 independent reflections
Radiation source: fine-focus sealed tube	2780 reflections with *I* > 2σ(*I*)
graphite	*R*_int_ = 0.019
φ and ω scans	θ_max_ = 30.0°, θ_min_ = 2.6°
Absorption correction: multi-scan (*SADABS*; Bruker, 2008)	*h* = −17→17
*T*_min_ = 0.57, *T*_max_ = 0.68	*k* = −28→28
11028 measured reflections	*l* = −12→12

### Refinement

**Table:**

Refinement on *F*^2^	Primary atom site location: structure-invariant direct methods
Least-squares matrix: full	Secondary atom site location: difference Fourier map
*R*[*F*^2^ > 2σ(*F*^2^)] = 0.022	Hydrogen site location: inferred from neighbouring sites
*wR*(*F*^2^) = 0.063	H-atom parameters constrained
*S* = 1.05	*w* = 1/[σ^2^(*F*_o_^2^) + (0.0348*P*)^2^ + 1.3062*P*] where *P* = (*F*_o_^2^ + 2*F*_c_^2^)/3
2881 reflections	(Δ/σ)_max_ = 0.001
129 parameters	Δρ_max_ = 0.42 e Å^−^^3^
0 restraints	Δρ_min_ = −0.34 e Å^−^^3^

### Special details

**Table:**

Experimental. Dark crystals from methanol. Bruker Kappa *APEX*II CCD diffractometer, full sphere data collection.
Geometry. All e.s.d.'s (except the e.s.d. in the dihedral angle between two l.s. planes) are estimated using the full covariance matrix. The cell e.s.d.'s are taken into account individually in the estimation of e.s.d.'s in distances, angles and torsion angles; correlations between e.s.d.'s in cell parameters are only used when they are defined by crystal symmetry. An approximate (isotropic) treatment of cell e.s.d.'s is used for estimating e.s.d.'s involving l.s. planes.
Refinement. Refinement of *F*^2^ against ALL reflections. The weighted *R*-factor *wR* and goodness of fit *S* are based on *F*^2^, conventional *R*-factors *R* are based on *F*, with *F* set to zero for negative *F*^2^. The threshold expression of *F*^2^ > σ(*F*^2^) is used only for calculating *R*-factors(gt) *etc*. and is not relevant to the choice of reflections for refinement. *R*-factors based on *F*^2^ are statistically about twice as large as those based on *F*, and *R*- factors based on ALL data will be even larger.

### Fractional atomic coordinates and isotropic or equivalent isotropic displacement parameters (Å^2^)

**Table:**

	*x*	*y*	*z*	*U*_iso_*/*U*_eq_	Occ. (<1)
Cu1	0.5000	0.285854 (8)	0.2500	0.01604 (6)	
O1	0.58573 (7)	0.35237 (3)	0.19375 (9)	0.01910 (14)	
N1	0.62650 (9)	0.21952 (4)	0.30042 (11)	0.01918 (16)	
C1	0.62862 (9)	0.44403 (5)	−0.00488 (13)	0.02073 (18)	
H1A	0.5465	0.4414	−0.0198	0.025*	
C2	0.66276 (10)	0.49606 (5)	−0.07553 (14)	0.0239 (2)	
H2A	0.6036	0.5284	−0.1396	0.029*	
C3	0.78315 (10)	0.50085 (5)	−0.05262 (13)	0.0227 (2)	
H3A	0.8060	0.5360	−0.1023	0.027*	
C4	0.87007 (10)	0.45401 (5)	0.04322 (13)	0.02263 (19)	
H4A	0.9529	0.4578	0.0615	0.027*	
C5	0.83576 (9)	0.40168 (5)	0.11223 (13)	0.02040 (18)	
H5A	0.8954	0.3696	0.1767	0.024*	
C6	0.71452 (9)	0.39567 (5)	0.08789 (12)	0.01713 (17)	
C7	0.67614 (9)	0.33962 (5)	0.16071 (11)	0.01684 (17)	
C8	0.73853 (9)	0.28017 (5)	0.18784 (13)	0.01919 (18)	
H8A	0.8005	0.2772	0.1522	0.023*	
C9	0.71826 (10)	0.22321 (5)	0.26408 (12)	0.01902 (18)	
C10	0.81019 (10)	0.16754 (5)	0.30347 (14)	0.0251 (2)	
H10A	0.7658	0.1263	0.2580	0.038*	
H10B	0.8598	0.1635	0.4250	0.038*	
H10C	0.8645	0.1768	0.2528	0.038*	
C11	0.60558 (11)	0.16066 (5)	0.37870 (13)	0.02317 (19)	
H11A	0.5853	0.1741	0.4689	0.028*	
H11B	0.6821	0.1341	0.4297	0.028*	
C12	0.5000	0.11873 (7)	0.2500	0.0241 (3)	
H12A	0.4673	0.0900	0.3093	0.029*	0.50
H12B	0.5327	0.0900	0.1907	0.029*	0.50

### Atomic displacement parameters (Å^2^)

**Table:**

	*U*^11^	*U*^22^	*U*^33^	*U*^12^	*U*^13^	*U*^23^
Cu1	0.01768 (9)	0.01421 (9)	0.01729 (9)	0.000	0.00895 (7)	0.000
O1	0.0193 (3)	0.0171 (3)	0.0243 (3)	0.0005 (2)	0.0130 (3)	0.0002 (2)
N1	0.0227 (4)	0.0162 (4)	0.0171 (4)	0.0019 (3)	0.0078 (3)	0.0007 (3)
C1	0.0175 (4)	0.0200 (4)	0.0242 (5)	−0.0008 (3)	0.0093 (4)	−0.0006 (3)
C2	0.0222 (5)	0.0208 (4)	0.0254 (5)	−0.0008 (4)	0.0083 (4)	0.0019 (4)
C3	0.0244 (5)	0.0227 (5)	0.0208 (4)	−0.0068 (4)	0.0103 (4)	−0.0020 (3)
C4	0.0192 (4)	0.0275 (5)	0.0225 (4)	−0.0055 (4)	0.0108 (4)	−0.0029 (4)
C5	0.0159 (4)	0.0240 (5)	0.0207 (4)	−0.0014 (3)	0.0079 (4)	−0.0008 (3)
C6	0.0164 (4)	0.0185 (4)	0.0164 (4)	−0.0019 (3)	0.0075 (3)	−0.0028 (3)
C7	0.0156 (4)	0.0184 (4)	0.0156 (4)	−0.0014 (3)	0.0064 (3)	−0.0026 (3)
C8	0.0174 (4)	0.0194 (4)	0.0205 (4)	0.0005 (3)	0.0085 (4)	−0.0031 (3)
C9	0.0193 (4)	0.0175 (4)	0.0161 (4)	0.0020 (3)	0.0046 (4)	−0.0033 (3)
C10	0.0238 (5)	0.0208 (5)	0.0260 (5)	0.0065 (4)	0.0075 (4)	−0.0027 (4)
C11	0.0308 (5)	0.0183 (4)	0.0199 (4)	0.0031 (4)	0.0113 (4)	0.0039 (3)
C12	0.0327 (8)	0.0159 (6)	0.0260 (7)	0.000	0.0154 (6)	0.000

### Geometric parameters (Å, °)

**Table:**

Cu1—O1	1.9145 (7)	C5—C6	1.3978 (13)
Cu1—O1^i^	1.9145 (7)	C5—H5A	0.9500
Cu1—N1	1.9394 (9)	C6—C7	1.4928 (13)
Cu1—N1^i^	1.9395 (9)	C7—C8	1.3882 (13)
O1—C7	1.2943 (11)	C8—C9	1.4241 (14)
N1—C9	1.3054 (14)	C8—H8A	0.9500
N1—C11	1.4688 (13)	C9—C10	1.5158 (14)
C1—C2	1.3925 (14)	C10—H10A	0.9800
C1—C6	1.3986 (14)	C10—H10B	0.9800
C1—H1A	0.9500	C10—H10C	0.9800
C2—C3	1.3900 (15)	C11—C12	1.5342 (14)
C2—H2A	0.9500	C11—H11A	0.9900
C3—C4	1.3903 (16)	C11—H11B	0.9900
C3—H3A	0.9500	C12—C11^i^	1.5342 (14)
C4—C5	1.3896 (14)	C12—H12A	0.9900
C4—H4A	0.9500	C12—H12B	0.9900
			
O1—Cu1—O1^i^	90.19 (4)	O1—C7—C8	126.18 (9)
O1—Cu1—N1	94.56 (3)	O1—C7—C6	114.90 (8)
O1^i^—Cu1—N1	154.41 (4)	C8—C7—C6	118.91 (9)
O1—Cu1—N1^i^	154.41 (4)	C7—C8—C9	126.09 (9)
O1^i^—Cu1—N1^i^	94.56 (3)	C7—C8—H8A	117.0
N1—Cu1—N1^i^	91.94 (5)	C9—C8—H8A	117.0
C7—O1—Cu1	123.15 (6)	N1—C9—C8	121.79 (9)
C9—N1—C11	121.70 (9)	N1—C9—C10	121.77 (9)
C9—N1—Cu1	125.72 (7)	C8—C9—C10	116.43 (9)
C11—N1—Cu1	112.51 (7)	C9—C10—H10A	109.5
C2—C1—C6	120.42 (9)	C9—C10—H10B	109.5
C2—C1—H1A	119.8	H10A—C10—H10B	109.5
C6—C1—H1A	119.8	C9—C10—H10C	109.5
C3—C2—C1	120.23 (10)	H10A—C10—H10C	109.5
C3—C2—H2A	119.9	H10B—C10—H10C	109.5
C1—C2—H2A	119.9	N1—C11—C12	111.17 (8)
C2—C3—C4	119.78 (10)	N1—C11—H11A	109.4
C2—C3—H3A	120.1	C12—C11—H11A	109.4
C4—C3—H3A	120.1	N1—C11—H11B	109.4
C5—C4—C3	120.01 (10)	C12—C11—H11B	109.4
C5—C4—H4A	120.0	H11A—C11—H11B	108.0
C3—C4—H4A	120.0	C11—C12—C11^i^	112.54 (12)
C4—C5—C6	120.79 (10)	C11—C12—H12A	109.1
C4—C5—H5A	119.6	C11^i^—C12—H12A	109.1
C6—C5—H5A	119.6	C11—C12—H12B	109.1
C5—C6—C1	118.73 (9)	C11^i^—C12—H12B	109.1
C5—C6—C7	121.42 (9)	H12A—C12—H12B	107.8
C1—C6—C7	119.84 (9)		
			
O1^i^—Cu1—O1—C7	171.40 (9)	Cu1—O1—C7—C8	−11.84 (13)
N1—Cu1—O1—C7	16.58 (8)	Cu1—O1—C7—C6	168.64 (6)
N1^i^—Cu1—O1—C7	−87.60 (11)	C5—C6—C7—O1	151.44 (9)
O1—Cu1—N1—C9	−12.71 (9)	C1—C6—C7—O1	−27.61 (13)
O1^i^—Cu1—N1—C9	−112.72 (10)	C5—C6—C7—C8	−28.11 (13)
N1^i^—Cu1—N1—C9	142.51 (10)	C1—C6—C7—C8	152.83 (9)
O1—Cu1—N1—C11	170.40 (7)	O1—C7—C8—C9	−3.07 (16)
O1^i^—Cu1—N1—C11	70.39 (11)	C6—C7—C8—C9	176.44 (9)
N1^i^—Cu1—N1—C11	−34.37 (6)	C11—N1—C9—C8	179.86 (9)
C6—C1—C2—C3	−0.83 (16)	Cu1—N1—C9—C8	3.24 (14)
C1—C2—C3—C4	−0.93 (16)	C11—N1—C9—C10	−1.20 (15)
C2—C3—C4—C5	1.64 (16)	Cu1—N1—C9—C10	−177.83 (7)
C3—C4—C5—C6	−0.59 (15)	C7—C8—C9—N1	7.75 (16)
C4—C5—C6—C1	−1.15 (15)	C7—C8—C9—C10	−171.24 (10)
C4—C5—C6—C7	179.79 (9)	C9—N1—C11—C12	−102.55 (11)
C2—C1—C6—C5	1.85 (15)	Cu1—N1—C11—C12	74.48 (9)
C2—C1—C6—C7	−179.07 (9)	N1—C11—C12—C11^i^	−37.68 (6)

Symmetry codes: (i) −*x*+1, *y*, −*z*+1/2.
